# Negative Impact of Skeletal Muscle Loss after Systemic Chemotherapy in Patients with Unresectable Colorectal Cancer

**DOI:** 10.1371/journal.pone.0129742

**Published:** 2015-06-12

**Authors:** Yuji Miyamoto, Yoshifumi Baba, Yasuo Sakamoto, Mayuko Ohuchi, Ryuma Tokunaga, Junji Kurashige, Yukiharu Hiyoshi, Shiro Iwagami, Naoya Yoshida, Masayuki Watanabe, Hideo Baba

**Affiliations:** 1 Department of Gastroenterological Surgery, Graduate School of Medical Sciences, Kumamoto University, Kumamoto, Japan; 2 Department of Gastroenterological Surgery, Cancer Institute Hospital of Japanese Foundation for Cancer Research, Tokyo, Japan; Hokkaido University, JAPAN

## Abstract

**Background:**

Skeletal muscle depletion (sarcopenia) is closely associated with limited physical ability and high mortality. This study evaluated the prognostic significance of skeletal muscle status before and after chemotherapy in patients with unresectable colorectal cancer (CRC).

**Methods:**

We conducted a retrospective analysis of 215 consecutive patients with unresectable CRC who underwent systemic chemotherapy. Skeletal muscle cross-sectional area was measured by computed tomography. We evaluated the prognostic value of skeletal muscle mass before chemotherapy and the rate of skeletal muscle change in cross-sectional area after chemotherapy.

**Results:**

One-hundred-eighty-two patients met our inclusion criteria. There were no significant differences in progression-free survival (PFS) or overall survival (OS) associated with skeletal muscle mass before chemotherapy. However, 22 patients with skeletal muscle loss (>5%) after chemotherapy showed significantly shorter PFS and OS compared with those without skeletal muscle loss (PFS, log-rank p = 0.029; OS, log-rank p = 0.009). Multivariate Cox regression analysis revealed that skeletal muscle loss after chemotherapy (hazard ratio, 2.079; 95% confidence interval, 1.194–3.619; p = 0.010) was independently associated with OS.

**Conclusions:**

Skeletal muscle loss after chemotherapy was an independent, negative prognostic factor in unresectable CRC.

## Introduction

Colorectal cancer (CRC) is the third leading cause of cancer deaths in Japan, with approximately 112,000 new cases and nearly 45,000 deaths in 2011 [1]. The past 20 years have seen remarkable progress in the treatment of CRC [2, 3]. However, its prognosis remains far from satisfactory.

Nutritional status and changes in body composition have been shown to affect perioperative surgical outcomes such as length of hospital stay and complication rates [4–6]. Skeletal muscle depletion (sarcopenia) is associated with increased toxicity from chemotherapy with 5-fluorouracil and its prodrug, capecitabine [7, 8]. Furthermore, sarcopenia is associated with poorer long-term outcomes in patients with gastrointestinal cancer, colorectal liver metastases, hepatocellular carcinoma, and melanoma [9–13]. In contrast, no consistent association between sarcopenia and survival has been demonstrated in pancreatic, oesophageal, or lung cancer [14–16].

We hypothesized that lower skeletal muscle mass before chemotherapy and skeletal muscle loss after chemotherapy might be associated with disease progression and higher mortality in patients with unresectable CRC. This study accordingly evaluated the association between skeletal muscle loss during chemotherapy and poor prognosis in patients with unresectable CRC.

## Patients & Methods

### Patients

From January 2005 to December 2013, we performed a retrospective analysis of 215 consecutive patients with unresectable CRC who had undergone systemic first-line chemotherapy at Kumamoto University Hospital (Kumamoto, Japan). Patients with unresectable, histologically confirmed colorectal adenocarcinoma were eligible for the study. Patients were included if they had received a computed tomography (CT) scan within 30 days before their first chemotherapy. Patients with a CT scan performed within 30–90 days from their first chemotherapy were eligible for evaluation of skeletal muscle changes. This retrospective study was approved by the institutional review board of Kumamoto University Hospital and was conducted in accordance with the Declaration of Helsinki. Prior written comprehensive informed consent for routine CT scan studies and treatment had been obtained from all patients, and IRB waived the requirement for additional informed consent to participate in this study. In addition, all identifiers were removed from our records at the completion of our analyses to protect patient privacy.

### Clinical data

We collected the following data from inpatient and outpatient records: relevant clinical data (age, sex, comorbidity and Eastern Cooperative Oncology Group performance status); tumour-specific data (location of primary tumour, number of organs with metastatic involvement and pre-treatment carcinoembryonic antigen [CEA] concentration); data concerning therapy (chemotherapy regimen); and overall response rate (ORR), progression-free survival (PFS), and overall survival (OS) data. We classified the primary tumour site as the right colon for tumours from the caecum through the transverse colon, and the left colon for tumours from the splenic flexure to the sigmoid colon and rectum.

Resectability was decided by a multidisciplinary team, including specialists in hepatic or colorectal surgery, during chemotherapy. There were no predefined criteria for resectability with regard to the number or size of the tumours, bilobarity, locoregional invasion, or presence of extrahepatic disease, though the resection needed to have the potential to be complete and macroscopically curative. The type of surgical resection was based on the results of preoperative and intraoperative diagnostic imaging. All detectable lesions were resected to achieve R0 resection.

### Measurement of skeletal muscle area

Skeletal muscle area was measured retrospectively on CT scans performed before chemotherapy and at initial routine CT scan up to 3 months after chemotherapy, at the level of the third lumbar vertebra (L3) in the inferior direction, with the patient in the supine position. Briefly, we measured pixels using a window width of −30 to 150 HU to delineate the muscle compartments and to compute their cross-sectional areas in cm^2^ using the Volume Analyzer Synapse Vincent 3D image analysis system (Fujifilm Medical, Tokyo, Japan) (S1 Fig). The cross-sectional area of muscle (cm^2^) at the L3 level computed from each image was normalized by the square of the height (m^2^) to obtain the skeletal muscle index (cm^2^/m^2^). The rate of skeletal muscle change (%) between pre-treatment CT scan and first routine CT scan after chemotherapy was calculated. All measurements and calculations were performed independently by two trained examiners (Y.M. and Y. S.) who were blinded to the clinical outcomes at the time of quantification. Lin’s concordance correlation coefficient was 0.940 (95% confidence interval [CI], 0.922–0.954) (S1 Table).

### Statistical analysis

Statistical analyses were performed using PASW Statistics 18 (SPSS Inc., Chicago, IL, USA), apart from classification and regression tree (CART) survival analysis, which was performed using the “party” package in R software ver.2.13.1 (http://cran.r-project.org/). All p values were two-sided. All data were expressed as mean ± standard deviation or median (range). Categorical variables were expressed as number (percentage). The p values for multiple hypothesis testing were adjusted by Bonferroni correction to p = 0.0042 (= 0.05/12). Mann–Whitney U and χ^2^ tests were used to compare groups and proportions between groups, respectively. Survival curves were estimated using the Kaplan–Meier method and analysed using log-rank tests. Univariate Cox proportional-hazards models of all potential baseline predictors were built to compute the hazard ratios (HRs) and their 95% CIs. We constructed a multivariate Cox proportional hazard model to compute the HR for skeletal muscle loss rate (<5% vs ≥5%), containing sex (male vs female), age at treatment (>70 vs ≤70 years), timing of metastases (simultaneous vs metachronous), primary tumour location (right colon vs left colon vs rectum), pre-treatment serum CEA level (>100 ng/ml vs ≤100 ng/ml), number of metastatic sites (1 vs ≥2), target agents (not combined vs combined), and R0 resection of metastatic lesions (no vs yes). A backward stepwise elimination with a threshold of p = 0.10 was used to select variables in the final model. The threshold p value for variable elimination was 0.05.

## Results

**Fig 1:**
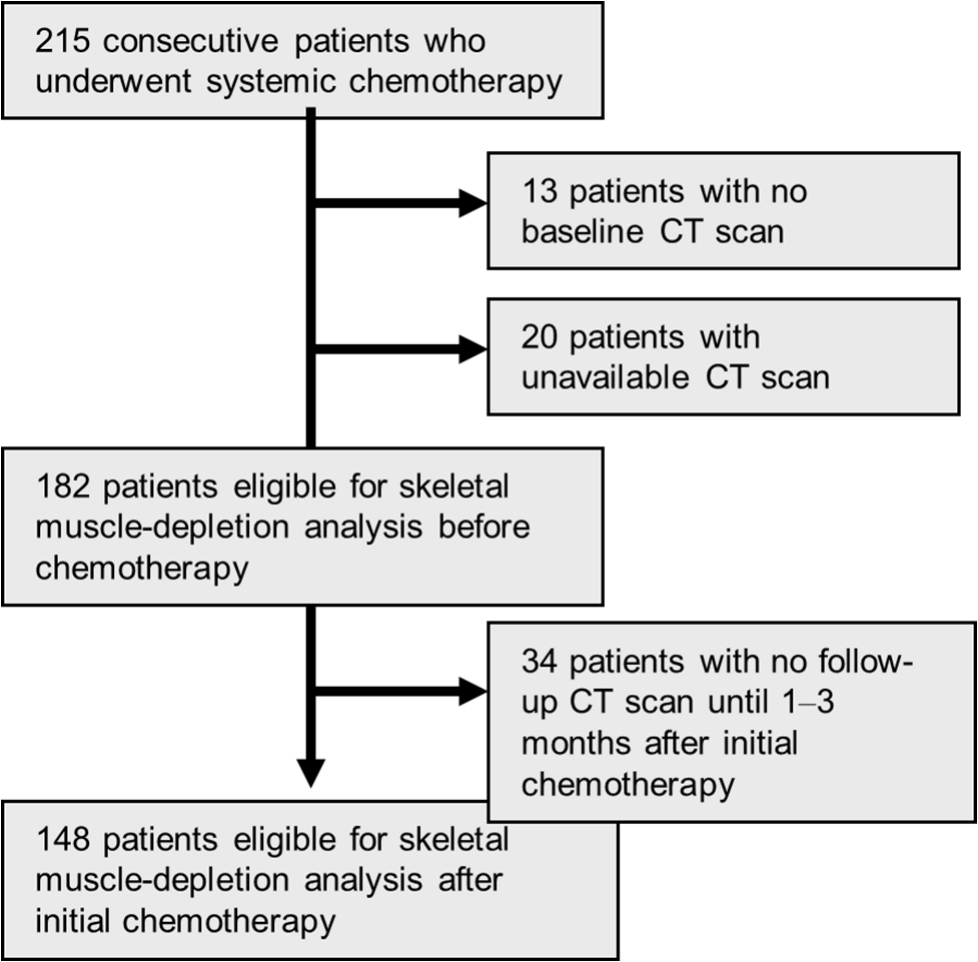
Flow chart representing the study selection process.

From our database of 215 patients with unresectable CRC, 182 patients (84.7%) met our inclusion criteria for analysis (Fig 1). The median follow-up periods for assessing PFS and OS were 8.1 months (range, 1–92 months) and 23.2 months (range, 1–100 months), respectively. During the follow-up period, 128 patients (70%) developed progression and 111 (61%) died.

**Fig 2:**
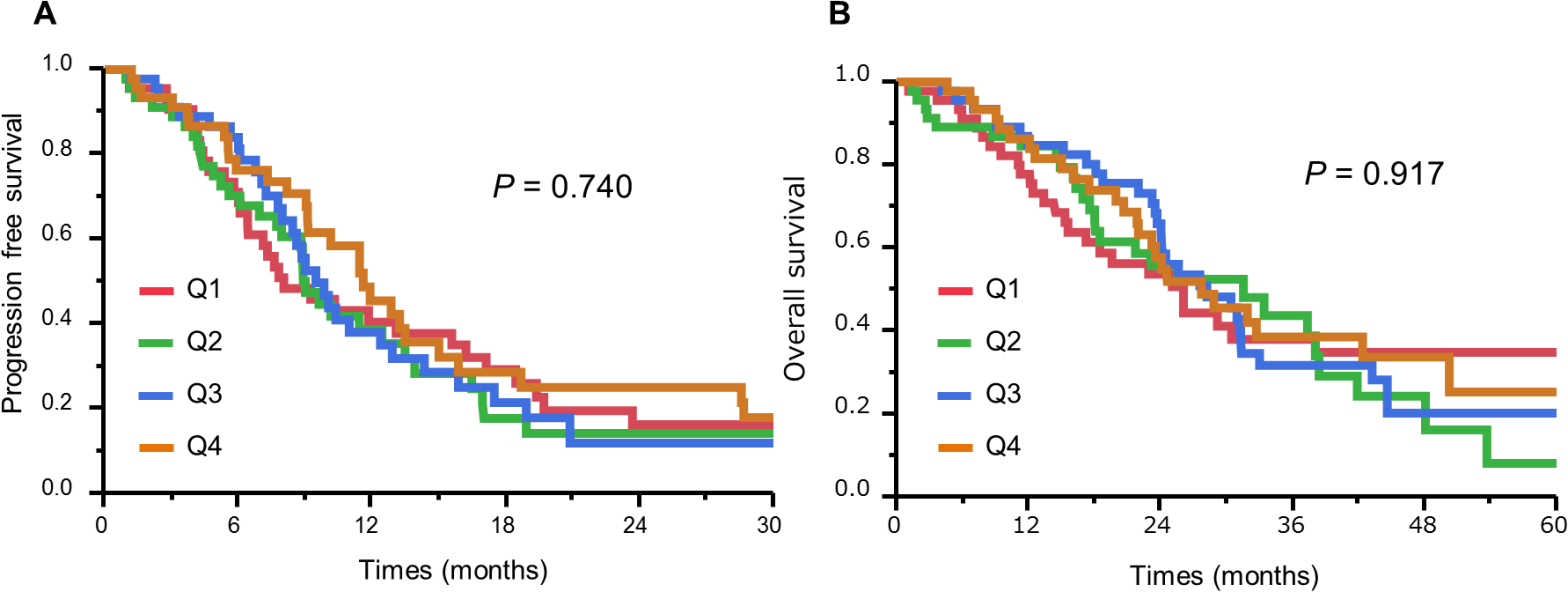
Kaplan–Meier curves of progression-free survival (A) and overall survival (B) in patients with unresectable colorectal cancer according to quartiles (Q1–Q4) based on skeletal muscle index before chemotherapy.

We examined the relationships between the skeletal muscle index in patients with unresectable CRC and various clinical and epidemiological variables. Female sex (p < 0.001) and BMI <25 (p < 0.001) were significantly associated with a lower skeletal muscle index (Table 1), though no other clinical CRC features were significantly correlated with skeletal muscle index. Cox regression analysis with skeletal muscle index before chemotherapy as a continuous variable showed no association between skeletal muscle index and increased mortality. Patients were divided into sex-specific quartiles according to pre-chemotherapy skeletal muscle index. Kaplan–Meier analysis found no significant difference in PFS (p = 0.740) or OS (p = 0.917) according to skeletal muscle index (Fig 2).

**Table 1:** Skeletal muscle index and clinical and tumour features in patients with unresectable colorectal cancer.

	n	(%)	Skeletal muscle index		p value
Age					0.971
<70	128	(70)	50.03	±8.46	
≥70	54	(54)	50.04	±8.46	
Sex					<0.001
Male	112	(62)	53.37	±7.70	
Female	70	(38)	45.00	±7.02	
PS					0.438
0–1	176	(97)	49.95	±8.37	
2	6	(3)	47.26	±6.92	
BMI					<0.001
<25	144	(79)	48.65	±7.67	
≥25	38	(21)	55.83	±9.06	
Location of primary					0.872
Right	48	(26)	50.46	±7.54	
Left	71	(39)	50.18	±9.05	
Rectum	63	(35)	49.89	±8.61	
Timing to metastases					0.108
Simultaneous	132	(73)	49.54	±8.24	
Metachronous	50	(27)	51.76	8.97	
Number of metastatic sites					0.403
1	97	(53)	50.66	±7.78	
≥2	85	(47)	49.57	±9.22	
Pre-treatment CEA level					0.266
≤100	102	(60)	50.99	±8.70	
>100	68	(40)	49.25	±7.60	
1st line chemotherapy					0.080
Oxaliplatin base	166	(91)	49.83	±8.53	
Irinotecan base	16	(9)	53.46	±7.41	
Combined target therapy					0.972
Not combined	74	(41)	50.05	±8.31	
Bevacizumab	82	(45)	50.31	±8.93	
Anti-EGFR antibody	26	(14)	49.93	±7.73	
Resection of metastases after chemotherapy					0.108
No	120	(66)	50.74	±8.49	
Yes	62	(34)	49.00	±8.39	

Abbreviations: CEA, carcinoembryonic antigen; EGFR, epidermal growth factor receptor.

Values given as mean ± standard deviation or number (%).

We also evaluated the association between changes in skeletal muscle mass after chemotherapy and survival. A total of 148 patients (68.8%) met our inclusion criteria for analysis (Fig 1). The median interval from first chemotherapy to initial CT scan after chemotherapy was 2.1 months (1.0–3.0). The median change in skeletal muscle was 4.2% (−30 to +39%) (S2 Fig). We found no correlation between the duration of chemotherapy (interval from first chemotherapy to initial CT scan after chemotherapy) and skeletal muscle change ratio (R2 = 0.012, p = 0.194) (S3 Fig).

We examined the relationship between the rate of skeletal muscle change and various clinical variables. None of the clinical CRC features was significantly correlated with the rate of skeletal muscle change (Table 2). We performed Cox regression analysis with the rate of skeletal muscle change as a continuous variable. Skeletal muscle change tended to be associated with increased overall mortality, but the effect was not significant (univariate analysis p = 0.083).

**Table 2:** Rate of skeletal muscle change after chemotherapy and clinical and tumour features in patients with unresectable colorectal cancer.

	n	(%)	Skeletal muscle change rate		p value
Age					0.353
<70	108	(73)	3.33	±10.68	
≥70	40	(27)	5.44	±9.41	
Sex					0.057
Male	85	(57)	2.57	±9.11	
Female	63	(43)	5.70	±11.68	
PS					0.799
0–1	143	(97)	3.86	±10.41	
2	5	(3)	4.89	±10.04	
BMI					0.824
<25	120	(81)	3.87	±10.75	
≥25	28	(19)	4.00	±8.66	
Location of primary					0.826
Right	40	(27)	3.55	±9.32	
Left	53	(36)	4.39	±13.03	
Rectum	55	(37)	3.68	±8.10	
Timing to metastases					0.092
Simultaneous	108	(73)	4.42	±10.38	
Metachronous	40	(27)	2.49	±10.30	
Number of metastatic sites					0.122
1	78	(53)	2.47	±8.14	
≥2	70	(47)	5.49	±12.24	
Pre-treatment CEA level					0.274
≤100	84	(59)	4.76	±9.66	
>100	59	(41)	2.72	±11.54	
1st line chemotherapy					0.550
Oxaliplatin base	166	(91)	3.89	±10.02	
Irinotecan base	16	(9)	4.02	±14.19	
Combined target therapy					0.238
Not combined	57	(39)	5.12	±10.16	
Bevacizumab	67	(45)	4.16	±9.61	
anti-EGFR antibody	24	(16)	0.28	±12.35	
Resection of metastases after chemotherapy					0.435
No	103	(70)	3.69	±11.24	
Yes	45	(30)	4.38	±8.09	

Abbreviations: CEA, carcinoembryonic antigen; EGFR, epidermal growth factor receptor.

Values given as mean ± standard deviation or number (%).

We then divided the 148 patients into four categories according to the rate of skeletal muscle change after chemotherapy: Q1 (>5%, n = 64), Q2 (0–5%, n = 35), Q3 (−5–0%, n = 27), and Q4 (<−5%, n = 22) and performed Cox regression analysis using categorical variables. According to univariate Cox regression analysis, Q4 patients had a significantly higher mortality than Q1 patients (p = 0.013; HR, 2.127; 95% CI, 1.179–3.703), whereas mortality in Q2 patients was similar to that in Q1 patients (p = 0.165 for Q2) (Table 3). Based on this analysis, we established a dichotomous skeletal muscle index variable, defining Q4 as the skeletal-muscle-loss group and combining Q1, Q2, and Q3 into the non-skeletal-muscle-loss group. Patient and tumour characteristics for both groups are summarized in Table 4. There was no significant difference between the two groups in any of the variables. CART survival analysis was used to validate the cut-off value for skeletal muscle change [17], and the first split point to partition the mortality risk for patients was a skeletal muscle change score of −3.6%. This result supports the validity of our cut-off value of ≥ −5.0%.

**Table 3:** Association between rate of skeletal muscle loss and mortality in patients with colorectal cancer.

		Univariate analysis		
Skeletal muscle change rate	Total n	HR	95% CI	p value
Q1 (>5%)	64	1.000		
Q2 (0–5%)	35	1.042	(0.590–1.786)	0.165
Q3 (−5–0%)	27	1.335	(0.741–2.320)	0.326
Q4 (<−5%)	22	2.127	(1.179–3.703)	0.013
Q1–3	126	1		
Q4	22	1.972	(1.140–3.23)	0.017

Abbreviations: HR, hazard ratio; CI, confidence interval.

**Table 4:** Characteristics of patients with initially unresectable colorectal cancer with or without skeletal muscle loss (>5%) during chemotherapy.

	Non-skeletal muscle-loss		Skeletal muscle-loss		p value
	n	(126)	n	(22)	
Age	64	(34–81)	60	(34–82)	0.242
Sex					0.269
Male	70	(56)	15	(68)	
Female	56	(44)	7	(32)	
Co-morbidity					0.896
Overall	68	(54)	11	(50)	0.522
Cardiovascular disorder	7	(6)	2	(9)	0.08
Respiratory disorder	2	(2)	1	(5)	0.364
Diabetes mellitus	21	(16)	1	(5)	0.140
BMI	22.2	(16.7–33.2)	21.7	(17.4–28.3)	0.996
Location of primary					0.310
Right	33	(26)	7	(32)	
Left	43	(34)	10	(45)	
Rectum	50	(40)	5	(23)	
Timing to metastases					0.583
Simultaneous	93	(74)	15	(68)	
Metachronous	33	(26)	7	(32)	
Number of metastatic sites					0.230
1	69	(55)	9	(41)	
≥2	57	(45)	13	(59)	
Pre-treatment CEA level					0.169
≥100	74	(61)	10	(45)	
<100	47	(39)	12	(55)	
1st line chemotherapy					0.855
Oxaliplatin base	116	(92)	20	(91)	
Irinotecan base	10	(8)	2	(9)	
Combined target therapy					0.015
Not combined	49	(39)	8	(36)	
Bevacizumab	61	(48)	8	(36)	
anti-EGFR antibody	16	(13)	6	(27)	
2nd line chemotherapy*					0.628
Done	92	(86)	18	(90)	
Undone	15	(14)	2	(10)	
ORR, %	20.5		16.7		0.713
PFS, months	6.8		4.7		0.355
3rd line chemotherapy^†^					0.074
Done	61	(62)	8	(40)	
Undone	38	(38)	12	(60)	
Resection of metastases after chemotherapy					0.064
Yes	42	(33)	3	(14)	
No	84	(67)	19	(86)	
Skeletal muscle status before chemotherapy					0.071
Non-sarcopenia	92	(73)	20	(91)	
Sarcopenia	34	(27)	2	(9)	

Abbreviations: CEA, carcinoembryonic antigen; EGFR, epidermal growth factor receptor.

*16 patients did not require 2nd line chemotherapy and 5 patients had missing information.

^†^22 patients did not require 3rd line chemotherapy and 7 patients had missing information.

Values given as median value (range) for age and BMI, or number (%).

**Fig 3:**
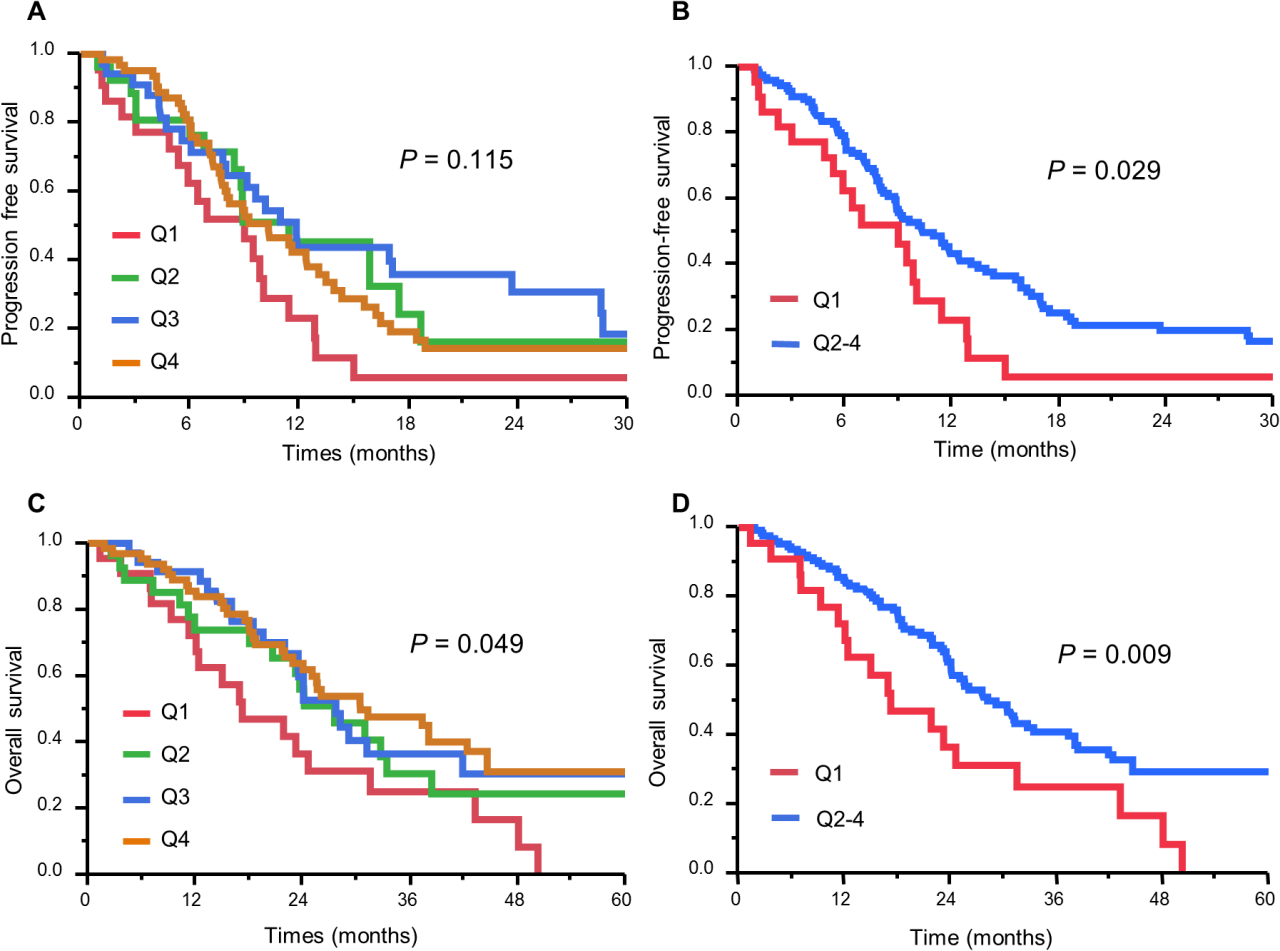
Analysis of survival in relation to change in skeletal muscle after chemotherapy in patients with unresectable colorectal cancer. Kaplan–Meier survival curves of progression-free survival (A) and overall survival (C) according to quartiles (Q1–Q4) based on change in skeletal muscle after chemotherapy in patients with unresectable colorectal cancer. (B) and (D) show differences in PFS and OS, respectively, between Q1 and Q2–4. Q1 represents the skeletal-muscle-loss group and Q2, Q3 and Q4 collectively represent the non-skeletal-muscle-loss group.

The ORRs were 41.9% in the skeletal-muscle-loss group and 51.5% in the non-skeletal-muscle-loss group. ORR was not significantly associated with skeletal muscle loss (p = 0.338). According to Kaplan–Meier analysis, patients in the skeletal-muscle-loss group (i.e., Q4 patients) experienced significantly shorter PFS (median PFS, 9.0 vs 10.3 months; log-rank p = 0.029) and OS (MST, 17.2 vs 28.2 months; log-rank p = 0.009) than those in the non-skeletal-muscle-loss group (Fig 3).

Cox multivariate modelling was used to determine whether skeletal muscle loss was independently associated with OS when known clinical prognostic markers were included. Univariate analysis initially identified sex, timing of metastases, pre-treatment CEA level, number of metastatic sites, R0 resection of metastatic lesions, and skeletal muscle loss as being associated with OS. Subsequent multivariate Cox analysis identified skeletal muscle loss as an independent risk factor for OS (HR, 2.079; 95% CI, 1.194–3.619; p = 0.010) (Table 5).

**Table 5:** Univariate and multivariate analyses of factors associated with overall survival in patients with skeletal muscle analysis.

Variable	Univariate analysis			Multivariate analysis		
	HR	95% CI	p value	HR	95% CI	p value
Skeletal muscle loss rate						
≤5%	1			1		
>5%	1.972	1.140–3.233	0.017	2.079	1.194–3.619	0.010
Age (years)						
<70	1					
≥70	1.274	0.800–1.974	0.299			
Sex						
Male	1					
Female	0.683	0.441–1.039	0.076			
Timing of metastases						
Simultaneous	1			1		
Metachronous	0.528	0.316–0.846	0.007	0.349	0.203–0.599	<0.001
Primary tumour location						
Right	1			1		
Left	0.544	0.328–0.904	0.019	0.406	0.242–0.681	0.001
Rectum	0.567	0.328–0.904	0.029	0.466	0.275–0.789	0.005
Pre-treatment CEA level, ng/ml						
≤100	1					
>100	1.838	1.213–2.779	0.004			
Number of metastatic sites						
1	1					
≥2	2.110	1.391–3.225	<0.001			
Target agents						
Not combined	1					
Combined	0.810	0.525–1.233	0.328			
Resection of metastases after chemotherapy						
No	1			1		
Yes	0.290	0.167–0.478	<0.001	0.276	0.161–0.474	<0.001

Abbreviations: CEA: carcinoembryonic antigen; HR, hazard ratio; CI, confidence interval.

## Discussion

In this study, we evaluated the association between skeletal muscle loss during chemotherapy and poor prognosis in patients with unresectable CRC. Pre-treatment skeletal muscle depletion was not a risk factor for survival in unresectable CRC. However, skeletal muscle loss (>5%) after chemotherapy was significantly associated with poorer PFS and OS.

Several studies have confirmed a close relationship between muscle wasting and poor prognosis in patients with various malignancies [11–13, 18]. In addition, accumulating evidence suggests that sarcopenia is linked to treatment toxicity in patients with various cancers [7, 19]. For example, Prado et al. [9] reported that sarcopenia was a significant predictor of toxicity and time to tumour progression in metastatic breast cancer patients treated with oral fluoropyrimidine [20]. In this study, however, there was no relationship between pre-treatment sarcopenia and tumour progression/survival in unresectable CRC patients. Sarcopenia may reflect the increased metabolic activity of a more aggressive tumour biology leading to systemic inflammation, causing muscle loss [21]. Achieving tumour control with effective chemotherapy therefore has the potential to reverse the catabolic processes causing cachexia.

The results of this study demonstrated that PFS in first-line chemotherapy and OS were significantly better in patients without skeletal muscle loss, compared with those with skeletal muscle loss. However, although the ORR was slightly lower in patients with skeletal muscle loss (42%) than in those without (52%), ORR was not significantly associated with skeletal muscle change (p = 0.338). The reason for this apparent discrepancy is unclear. However, the mechanisms underlying the negative impact of skeletal muscle loss on chemotherapeutic response and chemoresistance are likely to be multifactorial. In addition, the continuity of third-line or salvage chemotherapy was lower in patients with skeletal muscle loss (40%) compared with those without (62%) (p = 0.074). These data suggest that skeletal muscle loss may be associated with difficulties in continuing chemotherapy, and subsequent poor compliance might be a risk factor for a poor prognosis. Further studies are needed to verify the effects of skeletal muscle loss on the efficacy of chemotherapy.

There is currently limited knowledge of changes in muscle mass during chemotherapy. The present study found a median rate of change in skeletal muscle mass of 4.2% from pre- to post-chemotherapy, with almost two-thirds of patients remaining stable or gaining skeletal muscle mass. We confirmed that skeletal muscle mass began to change during the initial stage of chemotherapy. To the best of our knowledge, this study provides the first evidence for an association between skeletal muscle loss after chemotherapy and poor prognosis in patients with unresectable CRC. Stene et al. reported that skeletal muscle loss, but not sarcopenia at baseline, was a significant negative prognostic factor in patients with advanced non-small-cell lung cancer receiving palliative chemotherapy [22]. These results may confirm the value of skeletal muscle loss after chemotherapy as a prognostic factor in various cancers.

The most common definition of sarcopenia is currently an appendicular skeletal muscle index of more than two standard deviations below that of healthy adults (5.45 kg/m^2^ for women and 7.26 kg/m^2^ for men) [23]. However, these values are related to dual-energy X-ray scanning and we did not use this technique to obtain our primary measurements. Unlike dual-energy X-ray scanning, diagnostic CT is performed in all patients with unresectable CRC as part of routine examinations, and body-composition analysis using CT images is therefore readily available with no additional patient burden or cost, thus demonstrating wider clinical use. In this study, CART survival analysis calculated an optimal discriminatory cut-off value of 3.9% for skeletal muscle decrease, though we established 5% as a simple and reasonable cut-off value.

This study had some limitations. First, it was a single-institution, retrospective study with a small number of patients and a relatively short follow-up duration. Second, the definitions of both sarcopenia and skeletal muscle loss remain controversial. Nonetheless, our results provide important information relevant to the management of patients with unresectable CRC.

In conclusion, skeletal muscle loss after chemotherapy was an independent negative prognostic factor in unresectable CRC. Further prospective studies are needed to clarify the potential clinical benefits of these differences in patients with CRC.

## Supporting Information

S1 Fig An example of CT image analysis. a) We identified at the level of the third lumbar vertebra (L3) in the inferior direction, b) The skeletal muscle thresholds (-30 to +150HU) are applied, (c) the abdominal contents are cropped and the skeltal muscle cross sectional area calculated in cm^2^.
(TIF)

S2 Fig Waterfall chart of rates of skeletal muscle change in 148 cases. (TIF)

S3 Fig The correration between the skeletal muscle change ratio and the period from first chemotherapy. (TIF)

S1 Table Skeletal muscle area before chemotherapy measured by two examiners. (DOC)
