# Supplementary figures and images for: Negative Impact of Skeletal Muscle Loss after Systemic Chemotherapy in Patients with Unresectable Colorectal Cancer
Source: PLoS One. 2015 Jun 12;10(6):e0129742. doi: 10.1371/journal.pone.0129742 (PMC4466562; doi:10.1371/journal.pone.0129742)

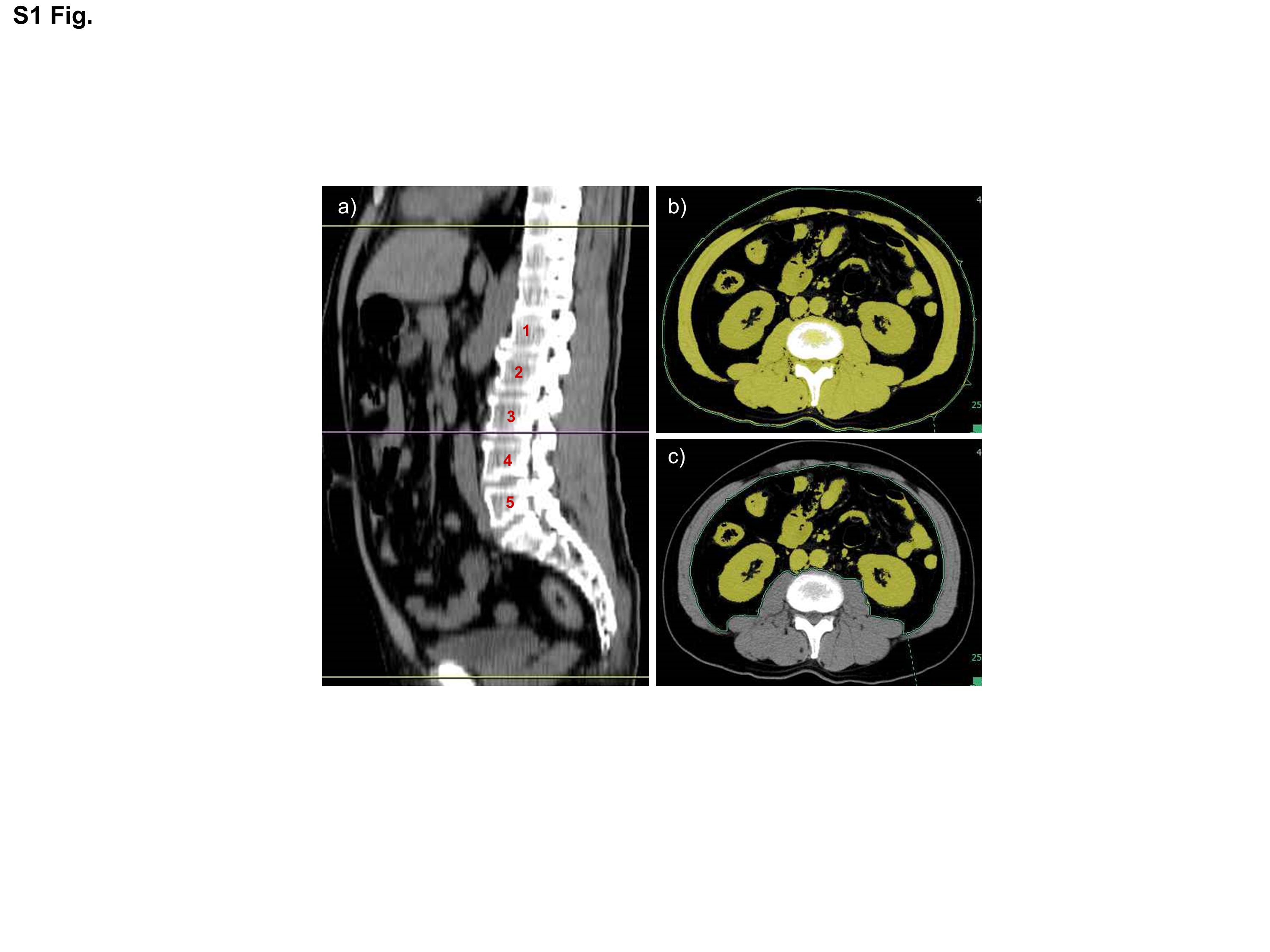

Supplement: S1 Fig — a) We identified at the level of the third lumbar vertebra (L3) in the inferior direction, b) The skeletal muscle thresholds (-30 to +150HU) are applied, (c) the abdominal contents are cropped and the skeltal muscle cross sectional area calculated in cm2. (TIF) [file pone.0129742.s001.tif]

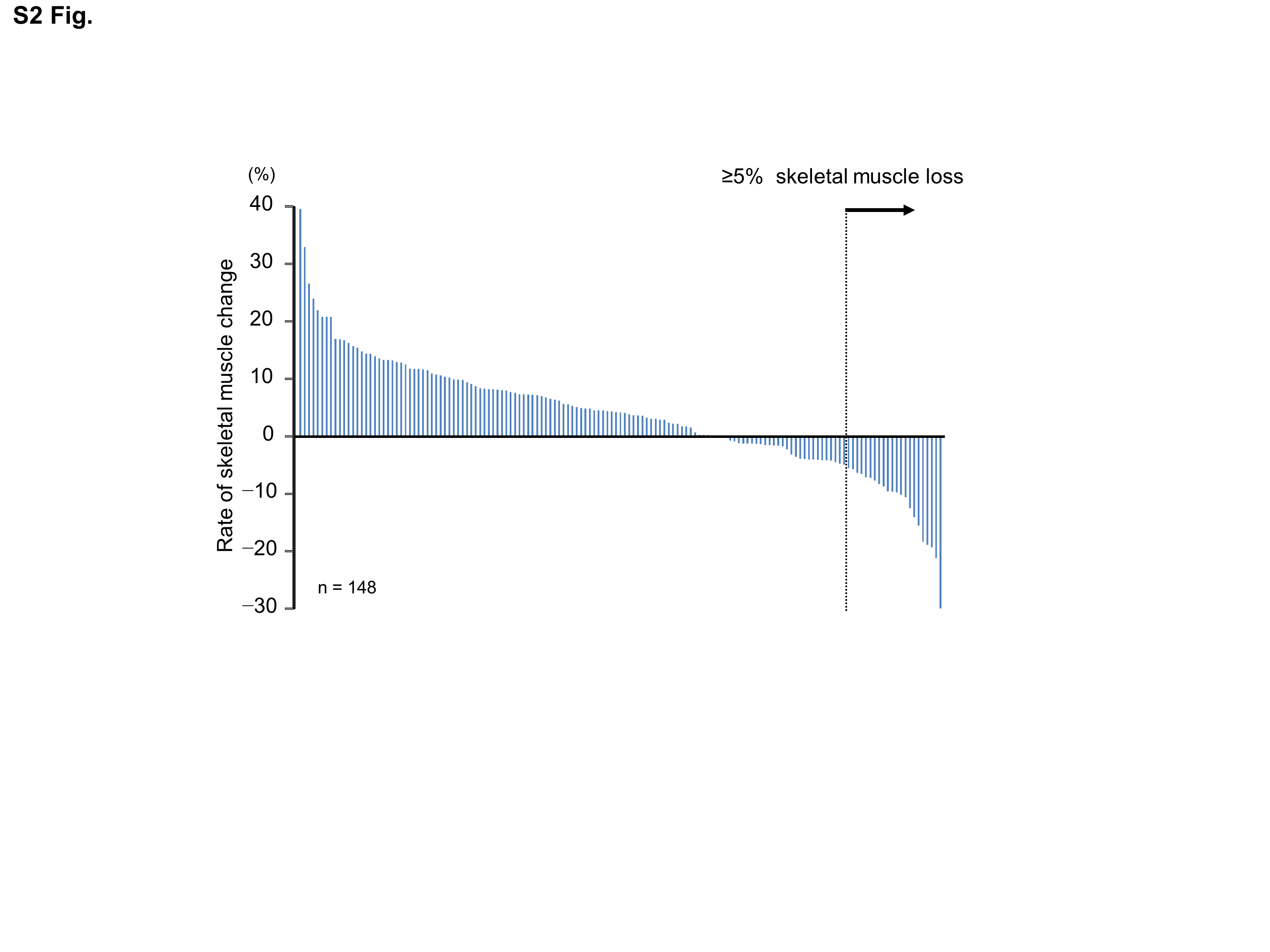

Supplement: S2 Fig — (TIF) [file pone.0129742.s002.tif]

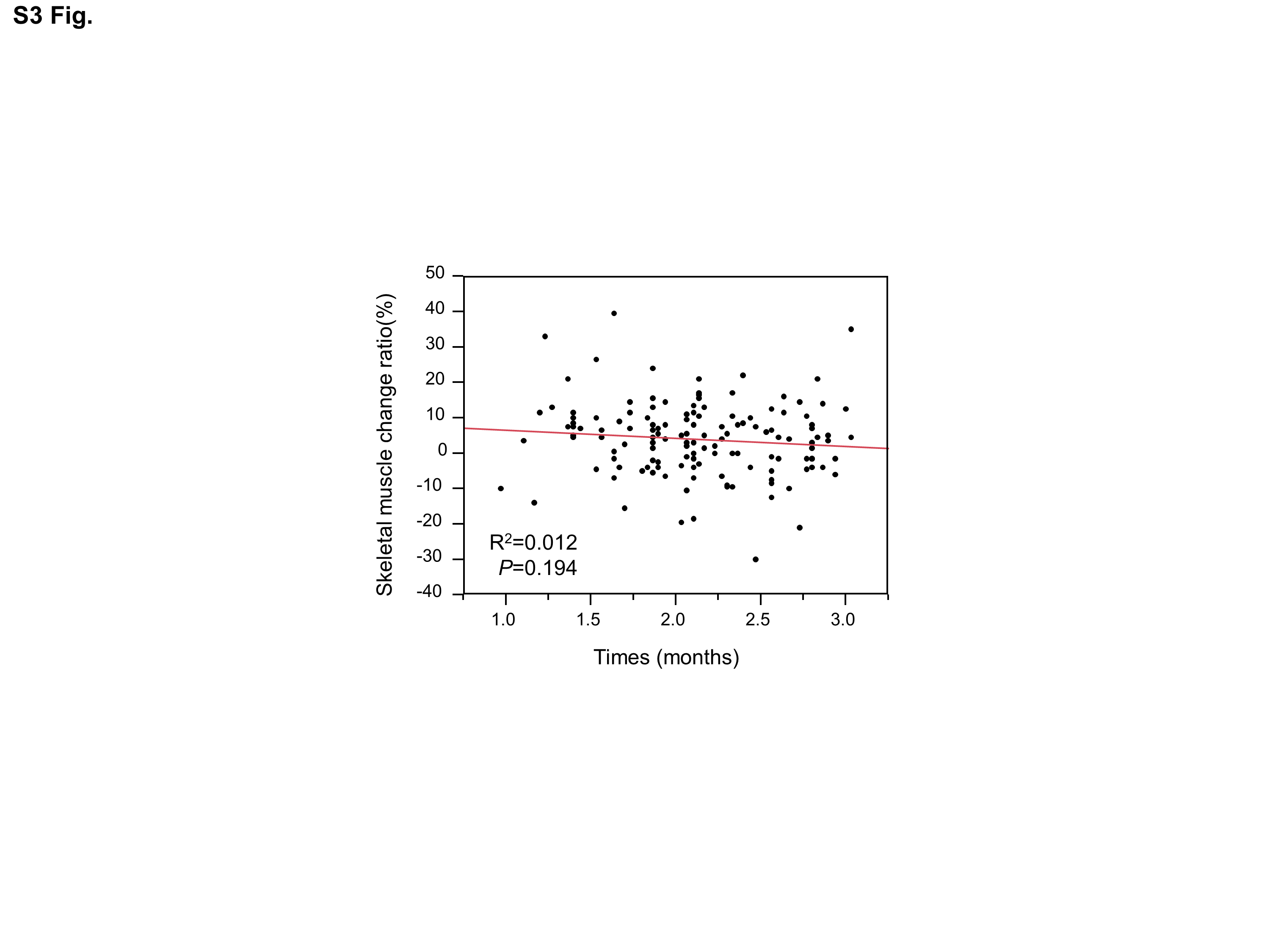

Supplement: S3 Fig — (TIF) [file pone.0129742.s003.tif]
