# Supplementary material for: Negative Impact of Skeletal Muscle Loss after Systemic Chemotherapy in Patients with Unresectable Colorectal Cancer
Source: PLoS One. 2015 Jun 12;10(6):e0129742. doi: 10.1371/journal.pone.0129742 (PMC4466562; doi:10.1371/journal.pone.0129742)
Supplement: S1 Table — (DOC) [file pone.0129742.s004.doc]

**Supplementary Table 2. Skeletal muscle area before chemotherapy measured by two examiners.**

| No | skeletal muscle area1  (cm^2^) | skeletal muscle area2  (cm^2^) |
| --- | --- | --- |
| 1 | 7455.78 | 7442.69 |
| 2 | 11681.57 | 10724.08 |
| 3 | 12002.22 | 8550.11 |
| 4 | 13092.00 | 10187.50 |
| 5 | 9911.91 | 9430.12 |
| 6 | 9599.30 | 8554.69 |
| 7 | 13540.31 | 11957.29 |
| 8 | 15195.54 | 13921.70 |
| 9 | 9791.35 | 10203.17 |
| 10 | 13963.62 | 12693.32 |
| 11 | 11012.87 | 9879.66 |
| 12 | 13050.06 | 12771.94 |
| 13 | 10102.57 | 10294.63 |
| 14 | 8619.12 | 8619.12 |
| 15 | 14214.82 | 12957.31 |
| 16 | 14823.03 | 13652.72 |
| 17 | 7734.02 | 7288.27 |
| 18 | 15546.62 | 15631.20 |
| 19 | 17021.93 | 15964.02 |
| 20 | 14851.27 | 13874.62 |
| 21 | 12211.96 | 11500.26 |
| 22 | 10405.85 | 9802.56 |
| 23 | 10617.68 | 9867.03 |
| 24 | 11916.16 | 12065.70 |
| 25 | 10496.03 | 9405.36 |
| 26 | 12994.69 | 12098.88 |
| 27 | 9759.98 | 9367.22 |
| 28 | 9090.86 | 8381.97 |
| 29 | 11622.80 | 11369.51 |
| 30 | 11886.09 | 11653.64 |
| 31 | 12130.19 | 13435.82 |
| 32 | 11590.57 | 10314.54 |
| 33 | 16910.20 | 15727.94 |
| 34 | 12438.00 | 13471.27 |
| 35 | 11977.71 | 10681.46 |
| 36 | 15112.36 | 14793.46 |
| 37 | 11470.89 | 9743.43 |
| 38 | 12896.56 | 12047.25 |
| 39 | 14853.50 | 14095.90 |
| 40 | 9292.65 | 8115.14 |
| 41 | 9518.44 | 9586.20 |
| 42 | 16490.10 | 15472.78 |
| 43 | 19936.17 | 18624.52 |
| 44 | 16311.81 | 16209.50 |
| 45 | 13954.48 | 12455.09 |
| 46 | 10934.71 | 11182.71 |
| 47 | 11150.78 | 9993.75 |
| 48 | 17387.90 | 16139.27 |
| 49 | 13682.60 | 13449.73 |
| 50 | 9104.92 | 9317.50 |
| 51 | 10360.93 | 9514.07 |
| 52 | 17922.82 | 14125.25 |
| 53 | 7998.73 | 9705.40 |
| 54 | 15237.28 | 14685.35 |
| 55 | 9347.30 | 9347.30 |
| 56 | 14893.67 | 13970.25 |
| 57 | 10302.81 | 9313.85 |
| 58 | 13384.38 | 12186.85 |
| 59 | 11469.73 | 11498.52 |
| 60 | 19214.03 | 18582.07 |
| 61 | 9518.48 | 9780.30 |
| 62 | 12407.83 | 12024.16 |
| 63 | 11822.42 | 11364.87 |
| 64 | 17999.17 | 17913.42 |
| 65 | 14506.88 | 13132.08 |
| 66 | 12385.01 | 9938.08 |
| 67 | 11513.26 | 11513.26 |
| 68 | 11134.51 | 11134.51 |
| 69 | 13306.38 | 12813.38 |
| 70 | 12685.34 | 11979.25 |
| 71 | 20077.46 | 19516.83 |
| 72 | 17408.82 | 16804.00 |
| 73 | 12108.12 | 11299.73 |
| 74 | 14826.57 | 13530.92 |
| 75 | 11171.28 | 9621.72 |
| 76 | 11282.95 | 10217.04 |
| 77 | 10455.00 | 9910.55 |
| 78 | 13691.74 | 12117.01 |
| 79 | 11561.95 | 10183.41 |
| 80 | 11340.92 | 10421.74 |
| 81 | 16250.84 | 15018.56 |
| 82 | 12779.26 | 12779.26 |
| 83 | 15144.65 | 14923.56 |
| 84 | 20440.07 | 19251.57 |
| 85 | 10869.87 | 10869.87 |
| 86 | 9346.05 | 11941.39 |
| 87 | 13755.57 | 13755.57 |
| 88 | 12265.91 | 12265.91 |
| 89 | 10480.10 | 10012.89 |
| 90 | 19119.60 | 18889.69 |
| 91 | 18391.12 | 15876.62 |
| 92 | 17990.60 | 16605.52 |
| 93 | 13656.86 | 12725.06 |
| 94 | 14201.26 | 12520.85 |
| 95 | 9107.22 | 8472.62 |
| 96 | 18826.46 | 17577.04 |
| 97 | 11991.87 | 11038.10 |
| 98 | 17504.19 | 16314.70 |
| 99 | 12540.94 | 11368.95 |
| 100 | 13570.91 | 13095.08 |
| 101 | 12255.11 | 12758.87 |
| 102 | 16181.42 | 15234.07 |
| 103 | 14297.35 | 13559.83 |
| 104 | 14459.22 | 13960.15 |
| 105 | 13913.66 | 12685.77 |
| 106 | 11905.08 | 11579.61 |
| 107 | 12166.01 | 11369.53 |
| 108 | 15912.71 | 15052.28 |
| 109 | 16005.04 | 15111.09 |
| 110 | 11225.95 | 9967.98 |
| 111 | 15798.49 | 15288.67 |
| 112 | 9103.48 | 8426.36 |
| 113 | 14406.87 | 12297.48 |
| 114 | 14782.58 | 14164.81 |
| 115 | 11795.59 | 10875.95 |
| 116 | 14306.25 | 14376.61 |
| 117 | 10015.24 | 8981.64 |
| 118 | 13893.30 | 13472.27 |
| 119 | 11516.46 | 11516.46 |
| 120 | 12571.32 | 12504.49 |
| 121 | 11983.45 | 12401.22 |
| 122 | 11628.77 | 11326.89 |
| 123 | 11783.36 | 11644.84 |
| 124 | 16748.66 | 15956.42 |
| 125 | 17304.45 | 16326.56 |
| 126 | 16481.86 | 17183.97 |
| 127 | 13182.13 | 13080.34 |
| 128 | 17327.97 | 15935.41 |
| 129 | 11014.89 | 9717.51 |
| 130 | 15139.55 | 15139.55 |
| 131 | 13180.67 | 13085.81 |
| 132 | 15434.01 | 15118.11 |
| 133 | 18399.72 | 17418.70 |
| 134 | 13106.85 | 12522.71 |
| 135 | 14159.67 | 13966.68 |
| 136 | 13051.70 | 13051.70 |
| 137 | 15192.61 | 14316.83 |
| 138 | 18371.15 | 17047.47 |
| 139 | 10858.67 | 9990.63 |
| 140 | 14556.89 | 14933.17 |
| 141 | 18695.50 | 18104.69 |
| 142 | 14287.19 | 12859.41 |
| 143 | 10401.12 | 9622.37 |
| 144 | 17059.31 | 15979.75 |
| 145 | 20039.71 | 16791.51 |
| 146 | 10107.24 | 9963.78 |
| 147 | 15701.30 | 15097.73 |
| 148 | 11513.26 | 10356.62 |
| 149 | 16207.38 | 14965.30 |
| 150 | 10070.33 | 10070.33 |
| 151 | 17747.07 | 17166.47 |
| 152 | 15006.86 | 15006.86 |
| 153 | 18525.66 | 17318.62 |
| 154 | 15022.63 | 14489.45 |
| 155 | 18459.77 | 18459.77 |
| 156 | 15828.94 | 16595.80 |
| 157 | 18231.73 | 18121.45 |
| 158 | 16176.99 | 14264.20 |
| 159 | 11403.79 | 10892.99 |
| 160 | 15701.29 | 16265.33 |
| 161 | 14447.53 | 15087.15 |
| 162 | 8762.11 | 8890.63 |
| 163 | 20422.98 | 18954.46 |
| 164 | 9513.15 | 8487.19 |
| 165 | 15261.57 | 15794.30 |
| 166 | 20880.12 | 19767.50 |
| 167 | 13144.23 | 12357.30 |
| 168 | 17263.95 | 17371.91 |
| 169 | 14914.78 | 14914.78 |
| 170 | 14024.48 | 13892.62 |
| 171 | 10516.17 | 10302.02 |
| 172 | 17135.07 | 16518.13 |
| 173 | 18042.29 | 18176.88 |
| 174 | 14915.67 | 16084.71 |
| 175 | 10863.16 | 10710.03 |
| 176 | 15588.39 | 14577.58 |
| 177 | 14358.28 | 13441.90 |
| 178 | 15953.61 | 15953.61 |
| 179 | 9028.53 | 9028.53 |
| 180 | 15674.74 | 15674.74 |
| 181 | 15751.60 | 14874.56 |
| 182 | 13772.28 | 12706.37 |
